# Fluorinated Graphene Prepared by Direct Fluorination of N, O-Doped Graphene Aerogel at Different Temperatures for Lithium Primary Batteries

**DOI:** 10.3390/ma11071072

**Published:** 2018-06-25

**Authors:** Xu Bi, Yanyan Li, Zhipeng Qiu, Chao Liu, Tong Zhou, Shuping Zhuo, Jin Zhou

**Affiliations:** 1 School of Chemistry and Chemical Engineering, Shandong University of Technology, Zibo 255049, China; bixu1991@163.com (X.B.); 18369904378@163.com (Y.L.); zpqiu78@163.com (Z.Q.); 15153327353@163.com (C.L.); zhuosp_academic@yahoo.com (S.Z.); 2 Lab of Functional Molecules and Materials, School of Physics and Optoelectronic Engineering, Shandong University of Technology, Zibo 255049, China

**Keywords:** fluorinated graphene, graphene, lithium primary battery, cathode material, fluorocarbon material

## Abstract

Fluorinated graphene (FG) has been a star material as a new derivative of graphene. In this paper, a series of fluorinated graphene materials are prepared by using N, O-doped graphene aerogel as precursor via a direct fluorination method, and the effect of fluorination temperature on the FG structure is investigated. The prepared FG samples are systematically characterized by scanning and transmission electron microscopy, X-ray photoelectron spectroscopy, X-ray diffraction, Fourier transform infrared spectroscopy, and Raman spectroscopy. It is found that the structure of FG, including features such as layer size, chemical composition, chemical bond state of the component elements, etc., is significantly related to the fluorination temperature. With the change of the fluorination temperature, fluorine atoms enter the graphene framework by a substitution process of the N, O-containing groups, including residual phenol, ether, carbonyl groups, or C–N groups, and the addition to CC bonds, subsequently forming a fluoride with different fluorine contents. The fluorine content increases as the fluorination temperature increases from 200 °C to 300 °C, but decreases at a fluorination temperature of 350 °C due to the decomposition of the fluorinated graphene. The prepared FG samples are used as cathode material for lithium primary batteries. The FG sample prepared at 300 °C gives a high specific capacity of 632 mAh g^−1^ and a discharge plateau of 2.35 V at a current density of 10 mA g^−1^, corresponding to a high energy density of 1485 Wh kg^−1^.

## 1. Introduction

Graphene, the world’s first two-dimensional (2D) material, was first isolated and characterized in 2004 by Andre Geim and Konstantin Novoselov at the University of Manchester. In simple terms, graphene is a thin layer of sp^2^ bonded carbon sheet. Graphene-based materials have attracted great attention in nanotechnology because of their amazingly attractive properties, such as high theoretical specific surface area (2630 m^2^ g^−1^), superior electron mobility, easy self-assembling into three-dimensional (3D) macroscopic materials with controlled microstructures, high mechanical, chemical, thermal, and electrochemical stabilities, etc. Therefore, graphene-based materials have already been widely applied in many fields, including but not limited to nanoelectronics [1], energy storage and conversion [2,3], sorption/separation [4], water purification [5], sensor [6], etc. However, pristine graphene suffers from several shortcomings, including a zero bandgap and structural defects chemical inertness. Therefore, enormous efforts have also been devoted to exploring graphene derivatives.

As a new derivative of graphene, fluorinated graphene (FG) could be regarded as the 2D basic structural element of fluorinated graphite, which was firstly prepared by Ruff in 1934 [7,8]. It not only retains the unique two-dimensional structure of graphene, but also exhibits different excellent properties due to the introduction of fluorine atoms, such as low surface energy, strong hydrophobicity and wide band gap [9]. At the same time, due to its extremely low surface energy, good chemical and thermal stability and high electromotive force, FG is considered to be promising, in applications like temperature-resistant, corrosion-resistant, abrasion-resistant, chemically stable high-temperature coatings with excellent lubricity, wear-resistant lubricating coatings and corrosion-resistant coatings [10,11]. Moreover, FG is regarded as an excellent cathode material for high-energy lithium fluorocarbon (Li/CF_x_) primary batteries [12,13,14]. The theoretical specific capacity of the Li/CF_x_ battery is determined by the F/C ratio, which could reach up to 865 mAh g^−1^ at x = 1, corresponding to an energy density of 2160 Wh kg^−1^ for a purely covalent C–F bond [15,16,17,18].

Similar to preparing graphene from graphite, FG could be obtained by exfoliating fluorinated graphite via mechanical or liquid exfoliation methods. Gong et al. prepared FG through a simple sonochemical exfoliation process in *N*-methyl-2-pyrrolidone (NMP), and the F/C ratio of FG can be facilely tuned just by adjusting the sonochemical time [19]. Sun et al. prepared high-quality FG by solvothermal exfoliation of fluorinated graphite through intercalation of acetonitrile and chloroform with low boiling points [12]. The prepared FG nanosheets exfoliated using chloroform showed a high specific capacity of 520 mAh g^−1^ and a voltage platform of 2.18 V at a discharge rate of 1 C. FG could also be prepared by fluorination of graphene or its derivatives. Using XeF_2_ as a fluorination resource, Nair et al. prepared FG and investigated its thermal and mechanical properties [20]. Several FG samples with various F/C ratio were synthesized by a direct fluorination procedure in an autoclave under a nitrogen/fluorine atmosphere at different exposure times and temperatures, and it was found that the F content significantly increased from 12.27 wt.% to 47.35 wt.% as the fluorination temperature increased from room temperature to 180 °C [21]. Although F_2_ is dangerous, the direct fluorination method by F_2_ still has many advantages compared to the liquid exfoliation methods and other fluorine resources (e.g., XeF_2_): this process can be easily scaled up, F_2_ has been large-scale produced by electrolysis technology, the F/C ratio can be finely controlled by varying the F_2_ concentration and fluorination temperature, and high F/C ratio can be easily obtained, which is very important for the cathode material of lithium primary batteries. 

Recently, hydrogels and aerogels prepared through reduction induced self-assembly of graphene oxides have attracted significant attention. These materials possess a three-dimensional (3D) porous structure, large specific surface area, and abundant heteroatom doping. These characteristics could ensure sufficient contact between the graphene and fluorine gas in the fluorination process, resulting in a homogeneous fluorination reaction and a high fluorination degree. However, the fluorination of graphene gels has not been reported. Herein, we prepared several FG materials via direct fluorination of N, O-doped graphene aerogel. The existence of N, O-containing groups strongly influence the fluorination process, and the fluorination result is very sensitive to the reaction temperature. With the increase in fluorination temperature, the F content firstly increased and then significantly decreased. The prepared FG materials are further investigated as cathode material for lithium primary battery. The specific discharge capacity increases and the discharge plateau decreases as the fluorine content increases. The FG-250 prepared at 250 °C showed a high discharge plateau about 3.0 V and a moderate discharge capacity of 512 mAh g^−1^ at a current density of 10 mA g^−1^, while the FG-300 prepared at 300 gives 632 mAh g^−1^ and 2.35 V.

## 2. Experimental

### 2.1. Preparation of Fluorinated Graphene

**Scheme 1:**
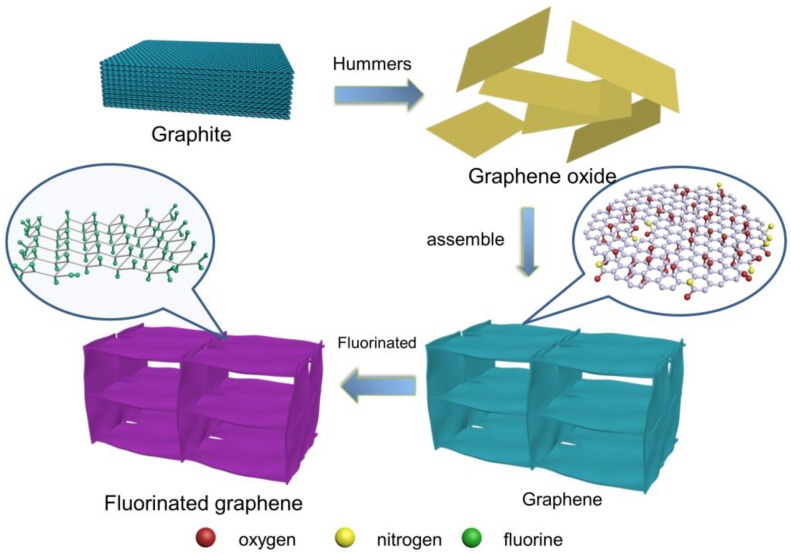
Illustration of the preparation of FG-x.

Graphene oxide (GO) was prepared from a natural flake graphite (Qingdao Huizhi graphite Co. Ltd., Qingdao, China) by Hummer’s method according to a previous report [22]. Afterward, a reduced graphene oxide aerogel (RGOA) was prepared according to Hu’s method [23]. In detail, 5 mL of GO solution (5 mg mL^−1^) was mixed with 20 μL of ethylenediamine (EDA) and then sealed in a Teflon reactor and heated for 6 h at 90 °C. The obtained graphene hydrogel was freeze-dried to convert it to a reduced graphene oxide aerogel. For the preparation of fluorinated graphene, the graphene aerogel was placed into a monel alloy tube and was heated up to the setting temperature at a rate of 2 °C min^−1^ under N_2_. Then, a mixed gas of F_2_/N_2_ (10 vol.% F_2_) was passed into the tube and kept at that temperature for 1 h. After the fluorination, the reactor was purged with N_2_ to remove the residual F_2_ and cooled to room temperature. The fluorinated products were thoroughly washed with deionized water to remove any unbounded fluorine, and vacuum-dried at 60 °C. For convenience, the fluorinated graphene was named FG-x, in which FG and x stand for fluorinated graphene and the fluorination temperature, respectively. The preparation process is illustrated in Scheme 1. *Caution, F_2_ is very dangerous, the fluorination experiments should be very carefully carried out, and the residual F_2_ should be adsorbed by charcoal!*

### 2.2. Materials Characterizations

The micro morphology of the prepared carbon materials was observed using a scanning electron microscope (SEM, Sirion 200 FEI, Eindhoven, Netherlands) and a transmission electron microscope (TEM, JEM2100, JEOL, Tokyo, Japan). Element composition and surface properties were characterized by energy dispersive spectroscopy (EDS, INCA Energy spectrometer, Eindhoven, Netherlands) and X-ray photoelectron spectroscopy (XPS, Escalab 250, Waltham, MA, USA), respectively. X-ray diffraction (XRD) patterns were conducted using Brucker D8 Advance diffraction with Cu Kαradiation (Brucker, Karlsruhe, Germany). Raman spectra were obtained using a LabRAM HR800 (Horiba, Kyoto, Japan) with the excitation wavelength set at 633 nm. Fourier transform infrared spectroscopy (FTIR) was conducted on a Nicolet 560 spectrometer (Thermo Fisher Scientific, Waltham, MA, USA).

### 2.3. Electrochemical Tests in Lithium Primary Battery

The performance of the prepared FG-x as cathode material for lithium primary batteries was investigated. The working electrode was prepared by mixing FG-x, Super P carbon, and a polyvinylidene fluoride (PVDF) binder with a weight ratio of 80:10:10 in *N*-methyl-2-pyrrolidinone (NMP). Then the ground slurry was coated onto an aluminum foil and dried in a vacuum oven at 80 °C for 24 h. The loading of active material on the electrode was about 1 mg cm^−2^. Coin-type test cells (CR2032) were fabricated with lithium foil working as both the anode and reference electrode, a microporous Celgard 2400 membrane as separator, and 1 M LiClO_4_ in a solution of PC-DME-DOL (Propylene carbonate-1,2-Dimethoxyethane-1,3-dioxolane) as electrolyte. The cells were assembled in a glove box under an argon atmosphere with concentrations of the moisture and O_2_ contents below 1 ppm. The button cells were discharged at different current densities on LAND CT2001A battery testing system with a discharge termination voltage of 1.5 V at room temperature.

## 3. Results and Discussion

**Figure 1:**
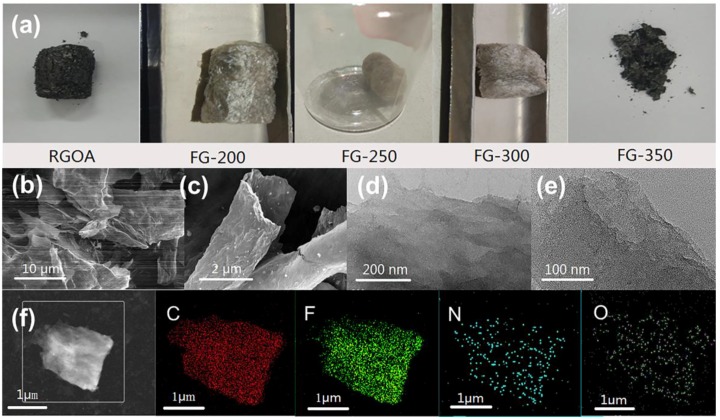
(**a**) Optical photographs of the synthesized RGOA, FG-200, FG-250, FG-300 and FG-350, from left to right, (**b**,**c**) SEM images of FG-250, (**d**,**e**) TEM images of FG-250, and (**f**) EDS mapping of FG-250.

Figure 1 and Figure S1 give the optical photographs of the synthesized samples of RGOA and FG-x and the images of SEM, TEM and EDS mapping. When it is treated hydrothermally, the GO is partially reduced by EDA, leading to the restoration of a portion of the sp^2^ regions; meanwhile, the nitrogen atoms could be doped into the graphene layers via EDA-mediated nucleophilic ring-opening reaction of epoxy groups and form graphene hydrogel with a typical 3D network structure through the π–π stack interaction in the hydrothermal reduction and doping process [22]. This 3D network structure ensures sufficient contact between the graphene and fluorine gas, rendering a homogeneous fluorination reaction. As shown in Figure 1a, as the temperature increased from 200 to 300 °C, the prepared FG-x samples still remained in the macroscopic shape of the freeze-dried RGOA, but with a loss of mechanical properties. Moreover, the color of the FG-x gradually became more grey white, indicating that the fluorine content of the FG-x was increasing. However, when the fluoridation reaction was raised to 350 °C, the aerogel structure collapsed and the color of FG-350 turned to dark brown (Figure 1a). This fact indicates that fluorination at 350 °C may be too intense, and the fluorine content decreases. As can be seen from the SEM images (Figure 1b,c), the FG-250 is an interconnected 3D structure consisting of randomly stacked multi-layered graphene sheets. Comparatively, the FG-350 layer has a clear fragmentation phenomenon, and the layer size of FG-350 is much lower than that of other FG-x samples (Figure S1a). This is related to the much harsher reaction conditions of high fluorination temperature. The RGOA and the fluorinated samples were further characterized using TEM observation. A typical 2D layered structure could be observed in all samples, and the edges of the prepared samples were transparent and wrinkled, with defined edges similar to soft silk. The chemical composition and element distribution of the FG-x were determined by EDS. As shown in Table S1, the fluorine ratios were determined to be 26.99%, 30.32%, 37.43% and 24.2% for FG-200, FG-250, FG-300, and FG-350, respectively, responding to F/C ratios of 0.43, 0.50, 0.67, and 0.51. Apparently, the fluorine content firstly increased and then significant decreased as the fluorination temperature increased. The oxygen and nitrogen contents showed the same change tendency, while the carbon content decreased monotonously. These facts will be explained below. The element map shows a uniform distribution of fluorine on the edges and the main body of the fluorinated samples, indicating a uniform reaction between RGOA and fluorine gas (Figure 1f and Figure S1c,d).

**Figure 2:**
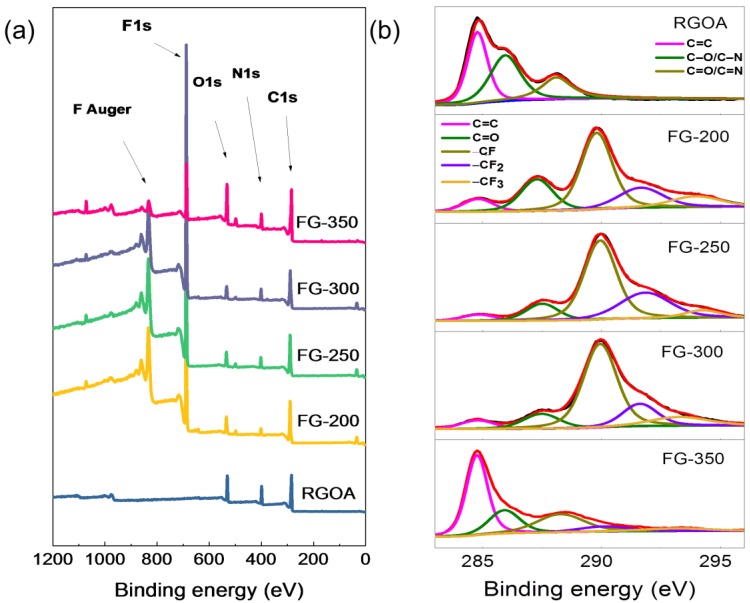
(**a**) XPS measurement spectra of RGOA and FG-x; (**b**) from top to bottom are the RGOA and FG-x high-resolution C1s spectra.

RGOA and the fluorinated samples were further analyzed by XPS to determine the elemental composition and properties of the chemical bonds in the FG-x. As shown in Figure 2a, the presence of F1s peaks (~690.0 eV) in all fluorinated samples indicates the successful fluorination of RGOA [24,25]. As seen in Table 1, the content of fluorine in FG-x increases with the increase of fluorination treatment temperature and reaches a maximum at 300 °C. Moreover, the contents of N, O elements significantly decrease after fluorination treatment. Except for FG-350, we found that the results of element composition were similar to those determined by EDS. For FG-350, the C content obtained by XPS was much higher than that obtained from EDS, while the converse situation was observed for the F content. Considering the difference in analysis depth between EDS and XPS, the above fact indicates that the F content in the bulk is higher than that on the surface or edge for this sample. In other words, some C–F bonds on the surface or edge decompose at higher temperatures (e.g., 350 °C). High-resolution XPS measurements were further conducted to analyze the chemical states of the C, F, N, and O species. As shown in Figure 2b, after fluorination, the peaks of C=C and C–O/C–N bonds suddenly decreased, and were transferred into fluorinated groups via additional reactions between C=C and fluorine radicals and the substitution reaction between N-or Ocontaining groups and fluorine radicals [26]. New components at about 289.9, 291.6, 293.3 eV were obtained after deconvolution in the C1s peak of all FG-x samples, demonstrating the existence of the –CF, –CF_2_ and–CF_3_ fractions (Figure 2b) [27]. The C–F bonds groups of CF_2_ and CF_3_ mainly formed at edges or at defect sites on the carbon surface. Obviously, FG-350 is special, for which the main C1s peak is the C=C bond (~284.8 eV), indicating that this sample still maintains a partial sp^2^ hybridization structure due to the decomposition of the C–F bonds into gaseous fluorocarbon (e.g., CF_4_) at this high temperature.

**Figure 3:**
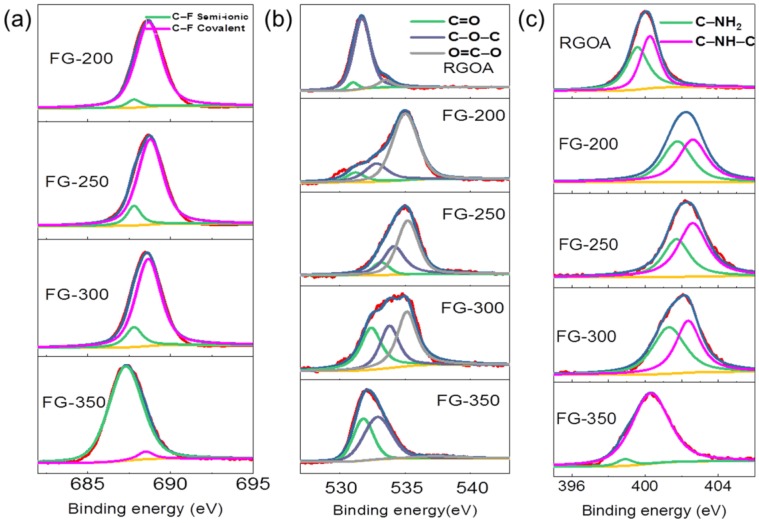
(**a**) FG F1s spectrum; (**b**) RGOA and FG O1s spectra; (**c**) RGOA and FG N1s spectra.

Figure 3 shows the F1s, O1s, and N1s spectra of the investigated samples. The F1s binding energies of the FG-x are located at 688.59 eV, 688.65 eV, 688.53 eV, and 687.33 eV for FG-200, FG-250, FG-300, and FG-350, respectively. Clearly, the binding energy of the F1s peak for FG-350 is much lower than those for other samples. After deconvolution, the F1s peak could be divided into two peaks at 688.6 eV and 687.5 eV, corresponding to covalent C–F bond and semi-ionic C–F bonds, respectively [12]. Such a shift down of position of the F1s peak is related to a change of the main nature of the C–F bond from covalent (sp^3^ hybridization of carbon atoms) to semi-ionic (sp^2^ hybridization of carbon atoms), as shown in Figure 3a. In other words, the partial sp^2^ hybridization structure of FG-350 results in a weakness of the intensity of its C–F bonds. From the O1s spectra of RGOA (Figure 3b), we could see that the oxygen-containing groups could be divided into a large number of C–O groups (hydroxyl groups or ether groups) and a few C=O bonds and O–C=O groups [28,29]. After fluorination, the peak of the C–O groups almost disappeared, while the C=O bond still remained, with a shift up to 535.2 eV resulting from the strong electronegativity of the F atom [30], indicating that the CO groups can be easily substituted to form C–F bonds. All of these observations show that O, N-containing functional groups are successfully converted to fluorinated units during the fluorination process. When fluorine gas interacts with RGOA, the hydroxyl/ether units could be converted to CF bonds via a F-induced nucleophilic substitution reaction, while the carbonyl and carboxyl units can be converted to –CF_2_ and –CF_3_ respectively. However, the concentration of CO groups increases when the fluorination temperature increases to 350 °C. The N1s spectra confirms the presence of amine groups after EDA-mediated assembly. These amine groups could be divided into C–NH_2_ (399.5 eV) and C–NH–C (400.3 eV), which are formed via the reaction between EDA and the oxygen-containing groups. The change of the N1s peak in Figure 3c is similar to the O1s. The possible reason may be that the fluorination reaction between the graphene and fluoride radical is too violent at high temperature, resulting in the loss of surface C atoms as a form of gaseous fluorocarbon. Actually, the weight of the fluorinated product is much lower than that of the RGOA resources, partially evidencing the above conjecture. However, the exact reason should be further investigated.

**Figure 4:**
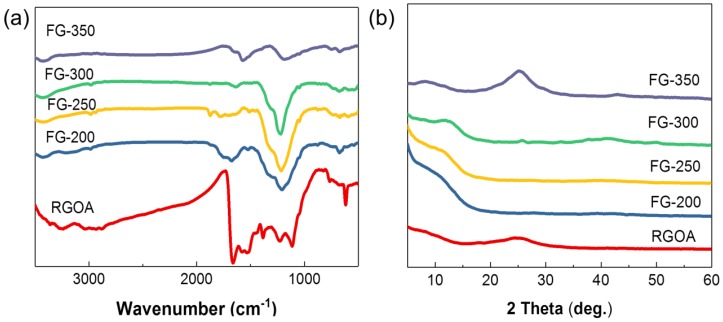
(**a**) FT-IR spectra and (**b**) XRD patterns of RGOA and FG-x.

The chemical structures of RGOA and FG were further studied by infrared spectroscopy (Figure 4a). It can be seen that RGOA has complicated absorption peaks at about 3241 cm^−1^, 1662 cm^−1^, 1532 cm^−1^ and 1114 cm^−1^, which are attributed to the stretching vibration peak of –OH, the C=O stretching vibrational peak, the bending vibration absorption peak belonging to carbonyl, and C–O–C vibration absorption peak, indicating the existence of –OH, –COOH, C–O–C, –C=O. Moreover, the peaks at about 1386 cm^−1^ and 1580 cm^−1^ are assigned to –CH_2_ and N–H groups. The formation of these groups can be attributed to the EDA-mediated nucleophilic ring-opening reaction of epoxy groups on the surface of GO according to the previous report [23]. After fluorination, the prepared FGs were thoroughly washed by water and vacuum-dried at 60 °C. Further considering the low boiling point of EDA (about 116 °C), the physically adsorbed EDA could be totally removed. The above facts indicate that the observed N atoms are incorporated into the RGOA structure. After fluoridation, all the above peaks of O, N-containing groups were significantly weakened, and a strong new band appeared at about 1215 cm^−1^ corresponding to the characteristic vibrational mode of C–F bond [31]. The exact C–F vibrational modes are 1213 cm^−1^, 1217 cm^−1^, 1226 cm^−1^, and 1190 cm^−1^ for FG-200, FG-250, FG-300, and FG-350, respectively. The C–F peak of FG-350 shows a significant red-shift, and the peak at 1572 cm^−1^ increased, further indicating the weakness of C–F bond and the increase of O content. The change trend exactly coincided with the XPS results. 

As shown in Figure 4b, the XRD pattern of RGOA shows a broad (001) diffraction peak at about 25° corresponding to an interplanar spacing of 0.37 nm, indicating the π–π stacking of the graphene sheets. This weak and broad XRD peak also reflects the poor ordering of graphene sheets along their stacking direction and the multi-layer stacked structure of the graphene sheets. The RGOA also shows a very weak XRD peak below 10°, corresponding to a larger layer spacing over 1 nm, which could be ascribed to the bridge link of EDA functionality and the large amount of oxygenated groups. After fluorination, the peak corresponding to the (001) reflection was found to shift to a much lower diffraction angle, indicating a broadening of the layer spacing. According to the Bragg equation, the layer spacing was calculated to be 0.90, 0.87, and 0.75 nm for FG-200, FG-250, and FG-300, respectively. As the temperature of fluorination increases, the substitution of fluorine for the N, O-containing groups is more complete, leading to a reduction of N, O-containing functional groups, resulting in a further decrease of interlayer spacing for FG-300. At 350 °C, the high temperature leads to intense reaction on the surface of carbon nano-sheets, and an in situ decomposition of the –CF_2_ and –CF_3_ groups on the surface, thus resulting in the partial recovery of π–π stacking of the graphene sheets for FG-350. Moreover, a weak peak at about 8.6°, corresponding to a large layer spacing of about 1.1 nm, was found for FG-350. This could be explained by the high content of N, O elements (the oxygenated groups and bridge-linked amide groups). The crystal structures of FG and RGOA were also characterized by the Raman spectra, which are shown in Figure S2. All samples show typical D and G peaks near 1350 cm^−1^ and 1590 cm^−1^, respectively. The presence of D peaks is a typical feature of chemically synthesized graphene. In general, the D peaks are closely related to the disorder in the graphene, and the G peaks are attributed to the first-order scattering of the stretching vibration mode E_2g_ observed for the sp^2^ carbon domain. A slight upshift of the G band from 1593 to 1605 cm^−1^ for the fluorinated samples was attributed to the formation of the C–F bond [21].

**Figure 5:**
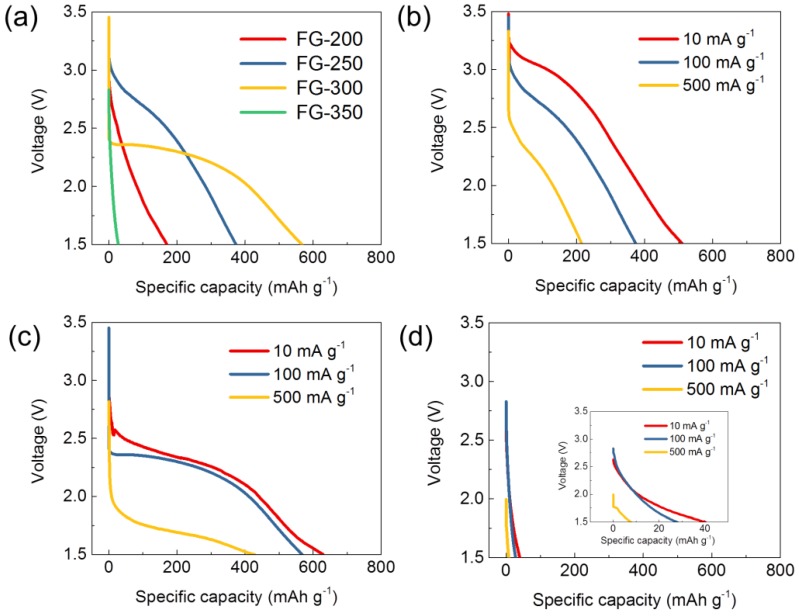
(**a**) Galvanostatic discharges curves of FG-x at 100 mA g^−1^, (**b**) galvanostatic discharge curves of FG-250, (**c**) galvanostatic discharge curves of FG-300, (**d**) galvanostatic discharge curves of FG-350 (inset is the enlarged curves).

The prepared fluorinated graphene materials were investigated as Li-primary battery cathode. Figure 5 shows the constant current discharge curve of FG-x. The maximum discharge capacity was obtained for a potential cut-off at 1.5 V vs. Li^+^/Li. As shown in Figure 5a, the cell voltage initially suddenly decreased, then remained a relatively constant level for FG-250 and FG-300 at 100 mA g^−1^. No discharge potential platform was observed for FG-200 and FG-350, which may be related to its low fluorine content [16]. The discharge capacities were 172 mAh g^−1^, 375 mAh g^−1^, 569 and 30 mAh g^−1^ for FG-200, FG-250, FG-300, and FG-350, respectively. These values are proportional to the fluorine content, but much lower than the theoretical ones according to the F/C ratio, since the fluorinated samples investigated here possess many –CF_2_ and –CF_3_ groups, which are proved to be non-electroactive [32]. Next, we studied the rate capability of the FG-200 and FG-300 in the range of 10 to 500 mA g^−1^. As shown in Figure 5b,c, the discharge capacity and voltage plateau decreased as the discharge current density increases. The FG-200 shows much higher discharge voltage plateau than FG-300 at all the current densities. This may be due to the lower fluorination degree resulting in higher conductivity between the fluorinated grains, reducing the cathodic overpotential. The discharge capacities of FG-300 were calculated to be 632 mAh g^−1^ and 430 mAh g^−1^ at 10 mA g^−1^ and 500 mAh g^−1^, corresponding to energy densities of 1485 Wh kg^−1^ and 731 Wh kg^−1^, respectively. It should be noted that there no apparent output potential delay was found at a high current density of 500 mA g^−1^ due to the large compact surface between the Li^+^ and the 2D fluorinated graphene. Finally, the discharge behavior of FG-350 was investigated, and the results are shown in Figure 5d. It could be seen that the cell voltage continuously decreased, and very low capacities were obtained at different current densities. The discharge curves of FG-350 are very similar to the initial of graphene as anode material for rechargeable lithium ion batteries, indicating that the capacities may be mainly ascribed to the adsorption of Li^+^ [33].

## 4. Conclusions

We have developed four fluorinated graphene materials by using a scalable method of direct fluorination. N, O co-doped fluorinated graphene nanosheets exhibit high fluorine content and amorphous microstructures with two-dimensional nanostructures. The structure of the fluorinated graphene is proved to be very sensitive to the fluorination temperature. As the fluorination temperature increases from 200 °C to 350 °C, the fluorine content firstly increases and then significantly decreases. The high fluorination temperature leads to a decomposition of C–F, thus resulting the loss of fluorine and partial restore of sp^2^ hybridization structure. Due to the large amount of oxygen groups in RGOA, CF_2_ and CF_3_ are produced during the fluorination process, which reduces the electrochemical performance of the fluorinated graphene as cathode materials of lithium primary batteries. The discharge capacity of FG-300 is calculated to be 632 mAh g^−1^ at 10 mA g^−1^, corresponding to energy densities of 1485 Wh kg^−1^, which are the highest ones among the investigated samples.

## Figures and Tables

**Table 1:** Surface element compositions determined by XPS.

Sample	C	F	O	N	C1s (%)					
					C=C	C–O (N)	C=O (N)	–CF	–CF_2_	–CF_3_
RGOA	67.5		16.1	16.4	39.3	39.3	21.4			
FG-200	62.3	26.5	6.1	5.2	5.8		10.9	57.1	15.8	10.3
FG-250	59.5	30.35	5.12	5.0	4.2		8.2	55.6	21.5	9.4
FG-300	54.8	35.6	4.7	4.9	7.5		7.9	56.1	17	11.2
FG-350	62.8	12.7	15.3	9.3	46.0		19.5	23.1	6.7	4.6

## Supplementary Materials

The following are available online at http://www.mdpi.com/1996-1944/11/7/1072/s1, Figure S1: (a) SEM images of RGOH, FG-200, FG-250, FG-300 and FG-350 from left to right; (b) TEM images of RGOH, FG-200, FG-250, FG-300 and FG-350 from left to right; (c) EDS images of FG-200; (d) EDS images of FG-300; (e) EDS images of FG-350, Figure S2: Raman spectra patterns of RGOA and FGHs, Table S1: Element component determined by EDS.
