# Supplementary material for: Fluorinated Graphene Prepared by Direct Fluorination of N, O-Doped Graphene Aerogel at Different Temperatures for Lithium Primary Batteries
Source: Materials (Basel). 2018 Jun 25;11(7):1072. doi: 10.3390/ma11071072 (PMC6073412; doi:10.3390/ma11071072)
Supplement: Supplementary file 1 [file materials-11-01072-s001.pdf]

materials

Supplement

Fluorinated Graphene Prepared by Direct Fluorination of N, O-Doped Graphene Aerogel at Different Temperatures for Lithium Primary Batteries

Xu Bi [1,†], Yanyan Li [1,†], Zhipeng Qiu [1], Chao Liu [1], Tong Zhou [2,*], Shuping Zhuo [1] and Jin Zhou [1,*]

[1] School of Chemistry and Chemical Engineering, Shandong University of Technology, Zibo 255049, China; bixu1991@163.com (X.B.); 18369904378@163.com (Y.L.); zpqiu78@163.com (Z.Q.); 15153327353@163.com (C.L.); zhuosp_academic@yahoo.com (S.Z.)

[2] Lab of Functional Molecules and Materials, School of Physics and Optoelectronic Engineering, Shandong University of Technology, Zibo 255049, China

* Correspondence: zhoutong@sdut.edu.cn (T.Z.); zhoujin@sdut.edu.cn (J.Z.)

† These authors do same contributions to this work.

Received: 14 May 2018; Accepted: 15 June 2018; Published: June 2018

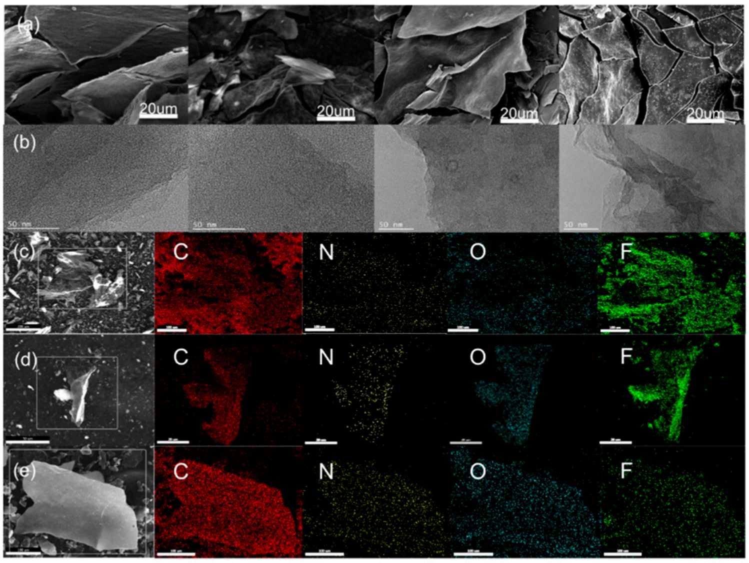

Figure S1. (**a**) SEM images of RGOH, FG-200, FG-250, FG-300 and FG-350 from left to right; (**b**) TEM images of RGOH, FG-200, FG-250, FG-300 and FG-350 from left to right; (**c**) EDS images of FG-200; (**d**) EDS images of FG-300; (**e**) EDS images of FG-350.

Table S1. Element component determined by EDS.

Sample	C	N	O	F	F/C
FG-200	62.48	4.09	6.43	26.99	0.43
FG-250	61.12	4.65	3.9	30.32	0.50
FG-300	55.77	3.59	3.21	37.43	0.67
FG-350	47.27	9.03	19.41	24.2	0.51

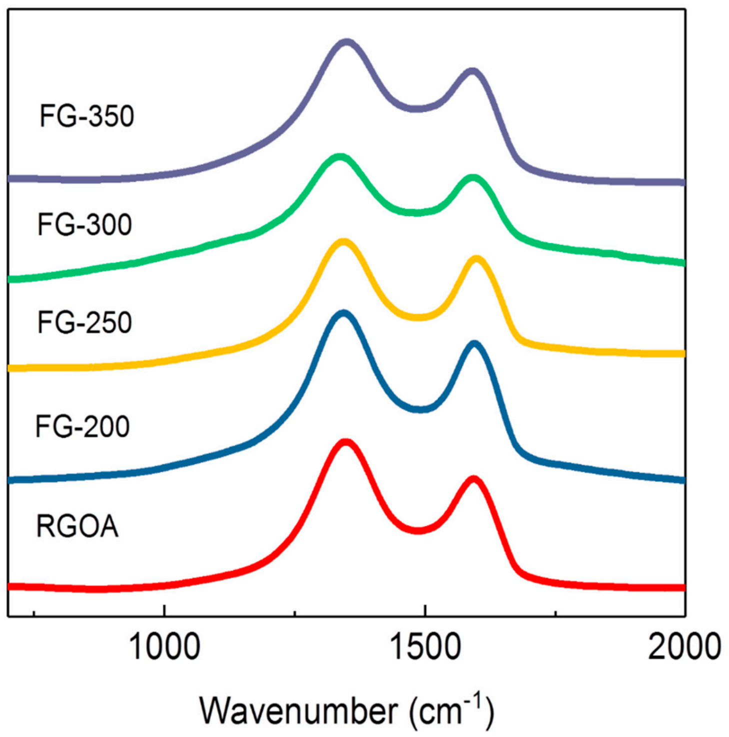

Figure S2. Raman spectra patterns of RGOA and FGHs.
